# ALKBH4 Functions as a Suppressor of Colorectal Cancer Metastasis via Competitively Binding to WDR5

**DOI:** 10.3389/fcell.2020.00293

**Published:** 2020-05-14

**Authors:** Chaoqin Shen, Tingting Yan, Tianying Tong, Debin Shi, Linlin Ren, Youwei Zhang, Xinyu Zhang, Yingying Cao, Yuqing Yan, Yanru Ma, Xiaoqiang Zhu, Xianglong Tian, Jing-Yuan Fang, Haoyan Chen, Linhua Ji, Jie Hong, Baoqin Xuan

**Affiliations:** ^1^State Key Laboratory of Oncogenes and Related Genes, Ren Ji Hospital, School of Medicine, Shanghai Jiao Tong University, Shanghai, China; ^2^Key Laboratory of Gastroenterology & Hepatology, Ministry of Health, Ren Ji Hospital, School of Medicine, Shanghai Jiao Tong University, Shanghai, China; ^3^Division of Gastroenterology and Hepatology, Ren Ji Hospital, School of Medicine, Shanghai Jiao Tong University, Shanghai, China; ^4^Shanghai Cancer Institute, Ren Ji Hospital, School of Medicine, Shanghai Jiao Tong University, Shanghai, China; ^5^Shanghai Institute of Digestive Disease, Ren Ji Hospital, School of Medicine, Shanghai Jiao Tong University, Shanghai, China; ^6^Department of Colorectal Surgery, Fudan University Shanghai Cancer Center, Shanghai, China; ^7^Department of Oncology, Shanghai Medical College, Fudan University, Shanghai, China; ^8^Department of Medical Oncology, Xuzhou Central Hospital, Xuzhou Medical University, Xuzhou, China; ^9^Department of Gastrointestinal Surgery, Ren Ji Hospital, School of Medicine, Shanghai Jiao Tong University, Shanghai, China

**Keywords:** CRC, EMT, epigenetic modification, metastasis, ALKBH4

## Abstract

**Background:**

Epithelial-Mesenchymal Transition (EMT) is a major process in the initiation of tumor metastasis, where cancer cells lose sessile epithelial potential and gain mesenchymal phenotype. Large-scale cell identity shifts are often orchestrated on an epigenetic level and the interplay between epigenetic factors and EMT progression was still largely unknown. In this study, we tried to identify candidate epigenetic factors that involved in EMT progression.

**Methods:**

Colorectal cancer (CRC) cells were transfected with an arrayed shRNA library targeting 384 genes involved in epigenetic modification. Candidate genes were identified by real-time PCR. Western blot, RNA-seq and gene set enrichment analysis were conducted to confirm the suppressive role of ALKBH4 in EMT. The clinical relevance of ALKBH4 in CRC was investigated in two independent Renji Cohorts and a microarray dataset (GSE21510) from GEO database. *In vitro* transwell assay and *in vivo* metastatic tumor model were performed to explore the biological function of ALKBH4 in the metastasis of CRC. Co-IP (Co-Immunoprecipitation) and ChIP (Chromatin Immunoprecipitation) assays were employed to uncover the mechanism.

**Results:**

We screened for candidate epigenetic factors that affected EMT process and identified ALKBH4 as a candidate EMT suppressor gene, which was significantly downregulated in CRC patients. Decreased level of ALKBH4 was associated with metastasis and predicted poor prognosis of CRC patients. Follow-up functional experiments illustrated overexpression of ALKBH4 inhibited the invasion ability of CRC cells *in vitro*, as well as their metastatic capability *in vivo*. Mechanistically, CO-IP and ChIP assays indicated that ALKBH4 competitively bound WDR5 (a key component of histone methyltransferase complex) and decreased H3K4me3 histone modification on the target genes including *MIR21*.

**Conclusions:**

This study illustrated that ALKBH4 may function as a novel metastasis suppressor of CRC, and inhibits H3K4me3 modification through binding WDR5 during EMT.

## Introduction

Colorectal cancer (CRC) is the second leading cause of cancer-related mortality worldwide (Bray et al., 2018). Despite the advance of treatment strategies involving surgery and medical therapy in CRC over the past decade, metastasis of CRC to distal sites is still the foremost cause of poor patients’ prognosis (Nishihara et al., 2013; Fidler and Kripke, 2015; Dekker and Sanduleanu, 2016). Nevertheless, the majority of CRC patients with distant metastasis are not appropriate candidates for conventional therapy and a paucity of effective clinical development exists for agents targeting the biological mechanisms underlying the metastatic process (Brenner et al., 2014). Consequently, identification and characterization of the molecular mechanism are imperative to facilitate the development of effective therapeutic strategies and biomarkers for CRC patients with metastasis.

Epithelial-Mesenchymal Transition (EMT) is a fundamental biological process in the initiation of tumor metastasis, which characterized as reversible loss of epithelial characteristics coupled with gain of mesenchymal properties (Thiery et al., 2009). Diverse lines of studies have revealed interesting links between EMT and epigenetic regulatory mechanisms. For instance, E-cadherin, which plays the essential role as a gatekeeper of the epithelial state in carcinomas (Hay, 1995; Vincent-Salomon and Thiery, 2003), has been discovered to be epigenetically suppressed. Moreover, it is becoming increasingly evident that the EMT is a high dynamic process that large-scale cell identity shifts are often orchestrated on a epigenetic level (Tam and Weinberg, 2013). However, the interplay between the modulation of epigenetic regulatory mechanisms and EMT remains poorly understood. Accordingly, although some molecular pathways explained the function of epigenetic factors in EMT have been partially elucidated, more straightforward targets and partners in the progression of EMT still need further exploration.

In recent years, RNAi technology has been well established as a useful tool for the construction of RNAi libraries and to reveal potential epigenetic markers modulating complex cellular processes at the genome-wide level (Westbrook et al., 2005a, b; Luo et al., 2008; Zender et al., 2008). Using an arrayed short hairpin RNA (shRNA) library targeting 384 genes involved in epigenetic modifications, we identified ALKBH4, a homolog of the *Escherichia coli* DNA demethylase AlkB family, as a suppressive modulator of EMT in CRC cells. In addition, low expression of ALKBH4 was associated with metastasis and poor prognosis in CRC patients and the biological function of ALKBH4 in CRC was also evaluated *in vitro* and *in vivo* models. Mechanically, ALKBH4 competitively bound WDR5 (a key component of histone methyltransferase complex) and decreased H3K4me3 histone modification on the target genes including *MIR21* and eventually prohibited EMT progression in CRC. Taken together, our study suggests that ALKBH4 is an upstream epigenetic inhibitor of EMT and may be a promising biomarker for CRC diagnosis and therapy.

## Materials and Methods

### Clinical Patient Specimen Collection

Tumor tissues and matched corresponding non-cancerous tissues were recruited from patients with CRC who underwent surgical resections at Department of Surgery, Renji Hospital Affiliated to Shanghai Jiao Tong University School of Medicine from December 2011 to March 2016. This study was approved by the Ethics Committee of Renji Hospital, Shanghai Jiao Tong University School of Medicine. Written informed consent was obtained from all participants before enrollment in this study. All the research was carried out in accordance with the provisions of the Declaration of Helsinki of 1975.

### Bioinformatics Analysis

CRC microarray datasets GSE21510 (Affymetrix Human Gene 1.0 ST Array) and their corresponding clinical data in this study were directly downloaded from Gene Expression Omnibus (GEO) database^1^. GSE21510 included 123 CRC samples and 25 non-tumor tissue samples.

### Cell Culture and Treatment

Human CRC cell lines HCT116, HT29, and SW480 were purchased from American Type Culture Collection (ATCC). All cell lines were cultured as recommended in growth medium supplemented with 10% fetal bovine serum (FBS, Gibco, United States) and incubated at 37°C with a humidified atmosphere of 5% CO_2_. ALKBH4 was overexpressed or knocked down by transduced with ALKBH4-overexpressing or ALKBH4 shRNA adenovirus, respectively. The vector was used as controls. Inhibition of miR-21 in cells were treated by miR-21 antagomir, and overexpression of miR-21 in cells were treated by miR-21 mimics (Genepharma, China).

### Western Blot

Western blot analysis was performed as described previously (Shen et al., 2017). Total protein was extracted from CRC cells using a total protein extraction buffer (Beyotime, China) containing a protease inhibitor mixture (protease inhibitors; phosphatase inhibitors; PMSF; KangChen, Shanghai, China). BCA Protein Assay Kit (Pierce Biotechnology) was used to measure the concentration of protein. Proteins were separated by 10–12% SDS-polyacrylamide gels and transferred to PVDF membranes (Millipore, Bedford, MA, United States). After blocked with 5% BSA at room temperature for 1.5 h, the membranes were incubated overnight with primary rabbit anti-ALKBH4(1:1000 dilution, Sigma, United States), rabbit anti-E-cadherin (1:1000 dilution, CST, United States), rabbit anti-fibronectin (1:1000 dilution, CST, United States), rabbit anti- ZO-1 (1:1000 dilution, CST, United States), rabbit anti-N-cadherin (1:1000 dilution, CST, United States), and GAPDH (1:1000 dilution, KangChen, China) antibodies at 4°C, and then washed with TBST for five times and incubated with species-specific secondary antibodies for 1 h at room temperature the next day. At last, the ECL detection system was used for visualization. Antibodies against GAPDH acted as an internal control.

### Immunohistochemical Staining

Human CRC tissue sections were rehydrated and treated with hydrogen peroxide for 15 min. Antigen retrieval was performed by microwave. After blocked with 10% normal goat serum for 30 min, the tissue microarray sections were incubated with ALKBH4 antibodies (1:200 dilution, Abcam, United Kingdom) on a humidified box at 4°C overnight. The next day, the sections were incubated with corresponding peroxidase-labeled secondary antibody for 30 min at room temperature and washed with PBS for three times. At last, Diaminobenzidine tetrahydrochloride (DAB; Maixin Biotech, China) was used for the color-reaction and hematoxylin was use for nucleus counterstaining. The immunohistochemical stained sections were observed under light microscopy.

Protein expression was assessed according to the intensity and extent of staining. The intensity of staining was evaluated on a scale of 0–3: 0, no staining; 1, weak staining; 2, moderate staining; 3, strong staining. The extent of ALKBH4 positive cells was assessed on a scale of 0–4: 0, 0–5%; 1, 6–25%; 2, 26–50%; 3, 51–75%; 4, 76–100%; A final score was obtained by using grades of the intensity staining × grades of extent. The tissues with a final score <6 were sorted into “ALKBH4 low expression” and those with a final score ≥6 were classified as “ALKBH4 high expression.”

### Total RNA Extraction and Quantitative Real-Time PCR

Total RNA was extracted from CRC cells, primary CRC tissues and adjacent non-cancerous tissues using Trizol reagent (Takara, Japan). Total RNA was reverse-transcribed using the PrimeScript RT Reagent Kit (Takara, Japan), and quantitative real-time PCR was performed using ABI reagent (Thermo Fisher Scientific, United States) by the StepOne real-time PCR system according to the manufacturer’s instruction. Primer sequences used in this study were listed as follows:

ALKBH4, forward, 5′-GGTCAGCCTCAACCTCCTGT-3′; reverse, 5′-TATCACGCTGTCC ACCAAGG-3′.
GAPDH, forward, 5′-GCATTGCCCTCAACGACCAC-3′; reverse, 5′-CCACCACCCTGTT GCTGTAG-3′.

### Transwell Invasion Assay

The transwell invasion assay were assessed using chambers (Millipore, United States). Initially, 2 × 10^5^ cells in serum-free medium were cultured in the upper chamber of a 24-well plate, and the corresponding medium supplemented with 20% FBS was placed in the lower chamber. After 48 h of incubation, the migrated cells were fixed with 4% paraformaldehyde for 20 min, stained with 0.1% crystal violet for 20 min, washed with PBS for five times, air dried and counted under a light microscope. Each experiment was repeated three times.

### Tumor Metastasis Model

Four-week-old male BALB/c nude mice were obtained from Experimental Animal Centre of Shanghai Laboratory Animal Center. HCT116 cells (5 × 10^6^ cells) were injected subcutaneously into the right flank of these mice to establish the CRC metastasis model. Seven days after subcutaneous inoculation, mice were randomly divided into different groups and were injected with PBS, control- overexpressing adenovirus or ALKBH4-overexpressing adenovirus by ways of multipoint intratumoral injection twice a week for 13 weeks. The mice were sacrificed at week 13. The numbers of lung metastatic foci were determined in H&E stained lung tissue sections under a binocular microscope (Leica, DM 300). All experimental procedures were approved by the Institutional Animal Care and Use Committee of Renji Hospital, Shanghai Jiao Tong University School of Medicine.

### Co-IP Assay

Coimmunoprecipitation was performed as described previously (Uyama et al., 2006). Briefly, HCT116 cells were harvested after indicated treatment. The whole-cell lysates were incubated with 2 μg of antibody or normal rabbit IgG at 4°C overnight, and two additional hours with 20 μl of 50% protein A agarose. Both input and IP samples were analyzed by western blot using various antibodies at the indicated dilutions: anti-WDR5 antibody (1:1000; Abcam), anti-H3K4me3 antibody (1:1000; CST) and anti- ALKBH4 (1:1000; Sigma).

### ChIP and High-Throughput Sequencing

Chromatin immunoprecipitation and high-throughput sequencing (ChIP-seq) was performed as follows. ChIP assays were conducted using the ChIP Assay Kit (Millipore, United States) according to the manufacturer’s protocols. HCT116 cells were seeded into 10 cm culture dish. Cells were cross-linked with formaldehyde and collected using SDS lysis buffer. The chromatin was sonicated to lengths between 200 and 1000 bp. The DNA-protein complexes were pre-cleared with Protein A Agarose/Salmon DNA and then immunoprecipitated with anti-WDR5 antibody, anti-H3K4me3 antibody and normal rabbit IgG. The co-precipitated DNAs were purified using phenol/chloroform and subjected to real-time PCR analysis. Library generation was performed using pooled ChIP DNA samples from three independent ChIP preparations using the Illumina protocol. Briefly, ChIP DNA fragment ends were repaired and phosphorylated using Klenow, T4 DNA polymerase and T4 polynucleotide kinase (Illumina kit components, United States). After ligation of Illumina adapters, DNA was size selected by gel purification and then PCR amplified using Illumina primers. Sequencing was performed at Genenergy Inc, Shanghai on an Illumina Hi-Seq 3000 machine. The FASTQ files were aligned to hg19 using Bowtie. Enriched regions were determined by the MACS program^2^ (Zhang et al., 2008) with a default setting.

### RNA Sequencing

ALKBH4 shRNA adenovirus was transduced into HT29 cells and high-throughput RNA sequencing was performed after knockdown of ALKBH4 in HT29 cells. For RNA sequencing of shRNA-transduced HT29 cells, each sample was cleaned up on a RNeasy Mini Column (Qiagen, Limburg, Netherlands), treated with DNase, and analyzed for quality on an Agilent 2100 Bioanalyzer. Samples were on an Illumina HiSeq 3000 for 2 × 150-bp paired-end sequencing. The RNAseq data analysis was performed according to the TopHat-HTSeq-DeSeq2 frame (Anders et al., 2013). Briefly, reads were mapped to the human genome (hg19) using TopHat v2.0.11^3^ (Kim et al., 2013) with the default options with a TopHat transcript index built from Ensembl_GRCh37. Count files of the aligned sequencing reads were generated by the htseq-count script from the Python package HTSeq with union mode, using the GTF annotation file (Anders et al., 2015). The read counts from each sequenced sample were combined into a count file, which was subsequently used for the differential expression analysis. Differential analyses were performed to the count files using DESeq2 packages, following standard normalization procedures (Love et al., 2014). Genes with <5 total counts in both conditions were removed from further analysis.

### RNAi Screening

Lentiviral shRNAs were arrayed for testing. Briefly, HT29 cells were transduced at a multiplicity of infection (MOI) of 0.3 in 96-well plate and real-time PCR was performed after 72 h.

### Statistical Analysis

All statistical analyses were carried out using R-3.0.2^4^. Correlation between ALKBH4 expression and clinicopathologic parameters in patients with CRC was examined by chi-square test. Overall survival was evaluated by Kaplan–Meier survival curve and analyzed by the log-rank test. Data from at least three independent experiments conducted in triplicates were presented as the mean ± SEM. The correlation of the two variables was examined by Spearman correlation test. Differences were considered to be significant with a value of *p* < 0.05.

## Results

### ALKBH4 Suppressed EMT in CRC

**FIGURE 1:**
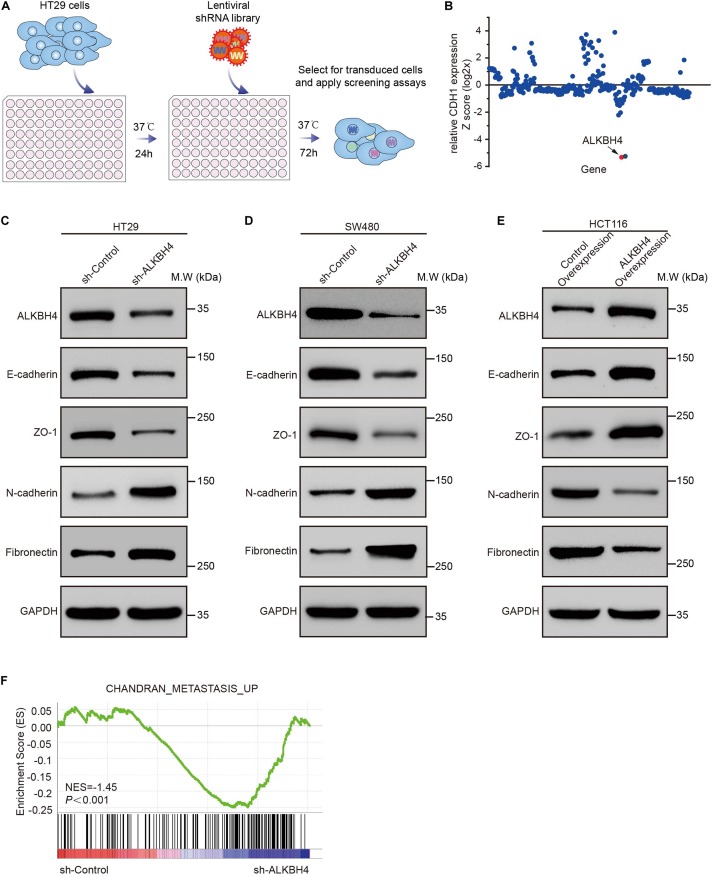
The suppressive role of ALKBH4 during EMT in CRC cells. **(A)** Schematic representation of the experimental workflow. HT29 cells were transduced with an arrayed lentiviral shRNA library targeting 384 genes involved in epigenetic modification. After 72 h transfection, RNA was extracted and real-time PCR was performed. **(B)** Dot plot of the lentiviral shRNA library screening result. The *y*-axis represents the *z*-scores for the relative CDH1 expression for each targeted gene. **(C,D)** Immunoblots of epithelial (E-cadherin and ZO-1) and mesenchymal (Fibronectin and N-cadherin) markers in HT29 and SW480 cells after ALKBH4 shRNA virus transduction, compared with control shRNA virus. GAPDH was used as a loading control. **(E)** Immunoblots of epithelial (E-cadherin and ZO-1) and mesenchymal (Fibronectin and N-cadherin) markers in HCT116 cells after transduced with ALKBH4-overexpressed virus and control- overexpressed virus. GAPDH was used as a loading control. **(F)** GSEA analysis of HT29 cells transduced with ALKBH4 shRNA virus.

For the purpose of identifying candidate epigenetic factors that involved in EMT progression, we performed an *in vitro* screening system using highly sensitive and quantitative lentiviral RNAi library. Briefly, human CRC cell line HT29, which exhibited higher expression of ALKBH4, was transduced with an arrayed lentiviral shRNA library targeting 384 genes involved in epigenetic modifications (Figure 1A and Supplementary Figure S1A). The expression of E-cadherin (encoded by the *CDH1* gene) was used as a screening criterion to select epigenetic genes involved in the EMT. Among all these candidate epigenetic genes, we discovered that knockdown of ALKBH4 has the most negative correlation with the relative expression of CDH1 in human CRC cells transduced with an arrayed shRNA library (Figure 1B). The results indicated that ALKBH4 might negatively regulate the progression of EMT in human CRC cells.

To confirm the suppressive role of ALKBH4 in EMT, Western blot assay was performed to detect the expression of both epithelial and mesenchymal molecular markers in ALKBH4-downregulated or ALKBH4-upregulated CRC cells. Obviously, downregulation of ALKBH4 significantly reduced the expression of epithelial markers, E-cadherin and zonula occludens-1 (ZO-1), and increased the expression of mesenchymal markers, Fibronectin and N-cadherin in HT29 and SW480 cells (Figures 1C,D). Conversely, overexpression of ALKBH4 significantly upregulated the expression of E-cadherin and ZO-1, and inhibited the expression of Fibronectin and N-cadherin in HCT116 cells (Figure 1E). Next, RNA sequencing (RNA-seq) analysis was performed to compare the gene expression profiles of CRC cells transduced with ALKBH4 shRNA and control shRNA. A total of 1157 downregulated genes and 3396 upregulated genes were detected after knockdown of ALKBH4 in CRC cells (Supplementary Table S1). Gene set enrichment analysis (GSEA) illustrated that “CHANDRAN_METASTASIS _UP” pathway was positively correlated with the downregulation of ALKBH4 in CRC cells (Figure 1F). These data strongly implicate that ALKBH4 may regulate EMT process in a suppressive way.

### Low Expression of ALKBH4 Is Clinically Related to Metastasis and Poor Prognosis in CRC

**FIGURE 2:**
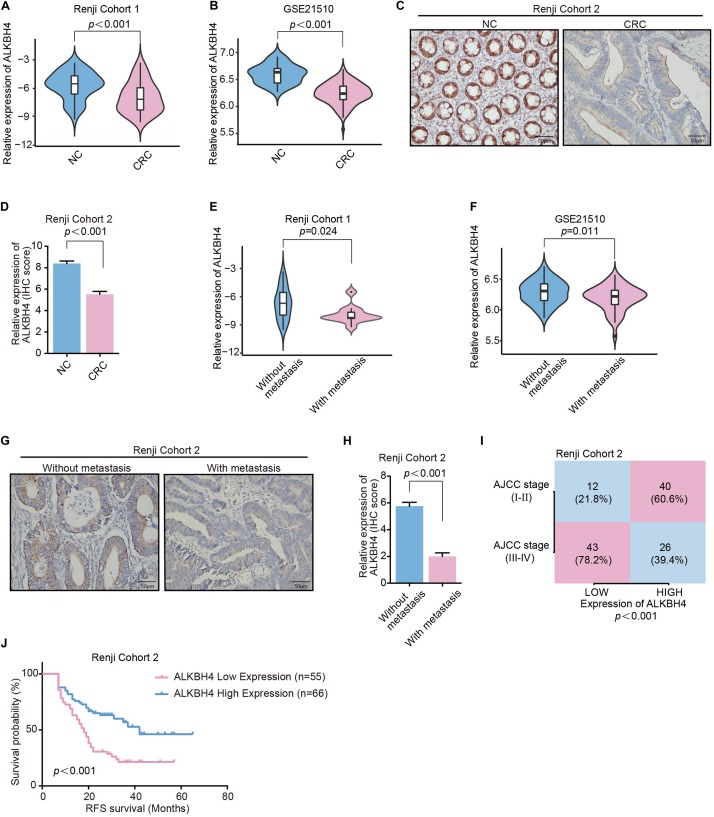
Downregulation of ALKBH4 correlates with metastasis and poor prognosis in CRC patients. **(A,B)** The relative expression of ALKBH4 in CRC tissues and adjacent non-tumor colorectal tissues were detected in Renji Cohort 1 **(A)** and dataset GSE21510 **(B)**, non-parametric Mann–Whitney test. **(C,D)** Representative immunohistochemistry images **(C)** and relative quantitative information **(D)** of CRC tissues and adjacent normal tissues for the protein levels of ALKBH4 in Renji Cohort 2, non-parametric Mann–Whitney test. **(E,F)** The relative expression of ALKBH4 in CRC tissues in patients with or without metastasis were detected in Renji Cohort 1 **(E)** and dataset GSE21510 **(F)**, non-parametric Mann–Whitney test. **(G,H)** Representative immunohistochemistry images **(G)** and relative quantitative information **(H)** of CRC tissues in patients with or without metastasis for the protein levels of ALKBH4 in Renji Cohort 2, non-parametric Mann–Whitney test. **(I)** Percentage of CRC patients with high expression and low expression of ALKBH4 stratified according to AJCC stage in Renji Cohort 2 (*n* = 121), Chi-square test. **(J)** Survival curves were generated using the Kaplan–Meier method and the log-rank test was used to evaluate the statistical significance of differences between CRC patients in Renji Cohort 2 with High or Low ALKBH4 expression, Log-rank test. Error bars in the scatter plots represent SEM.

It is documented that EMT is a critical event involved in the metastasis of CRC (Cao et al., 2015). Accordingly, we further analyzed the clinical significance of ALKBH4 in human samples. Real-time PCR revealed that the mRNA expression of ALKBH4 was significantly decreased in CRC tissues compared with paired adjacent non-tumor tissues from patients in Renji Cohort 1 (Figure 2A and Supplementary Table S2). Moreover, similar results can be counted in the microarray dataset (GSE21510) from GEO database that the mRNA expression of ALKBH4 was markedly decreased in human CRC tissues than in normal tissues (Figure 2B). Then, to examine endogenous ALKBH4 protein expression, we performed immunohistochemistry staining (IHC) on paraffin-embedded CRC tissues. The results showed a dramatically decreased expression of ALKBH4 in tumor tissues than paired non-tumor tissues from patients in Renji cohort 2 (Figures 2C,D and Supplementary Table S3).

Furthermore, the association between ALKBH4 expression and clinicopathological characteristics was analyzed. Analysis of the primary tumor tissue showed that patients with distant metastasis exhibited lower mRNA expression of ALKBH4 than those without metastasis both in Renji Cohort1 (Figure 2E) and microarray dataset (GSE21510) from GEO database (Figure 2F). IHC staining also confirmed that CRC patients with metastasis had a significantly lower expression of ALKBH4 (Figures 2G,H). In addition, ALKBH4 expression was negatively correlated with America Joint Committee on Cancer (AJCC) stage (Figure 2I). Kaplan–Meier analysis showed that CRC patients with low ALKBH4 expression had significantly shorter recurrence-free survival time than CRC patients with high ALKBH4 expression (Figure 2J). Collectively, these results indicate that low expression of ALKBH4 is clinically associated with metastasis and poor prognosis in CRC patients.

### ALKBH4 Inhibits Invasion *in vitro* and Metastasis *in vivo* in CRC

**FIGURE 3:**
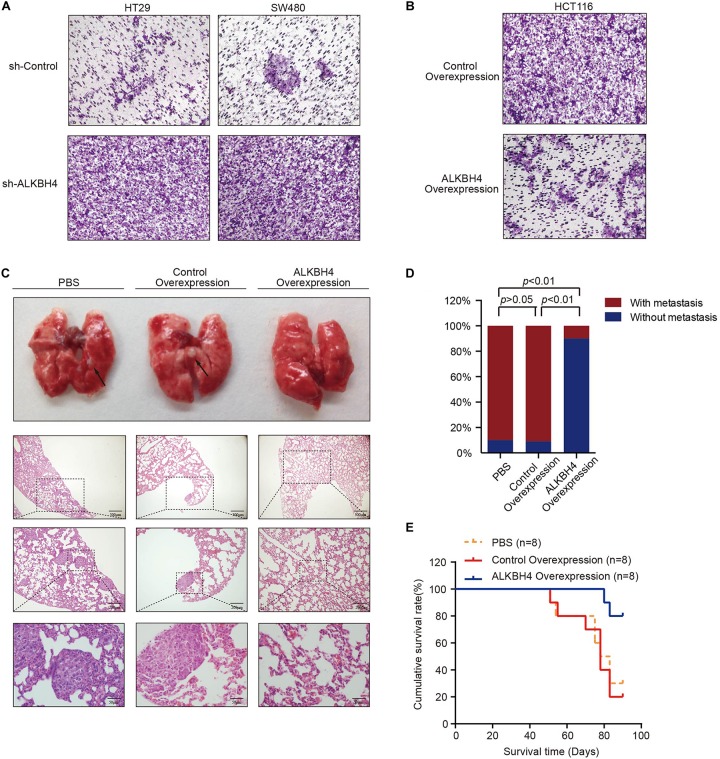
ALKBH4 inhibits cell invasion *in vitro* and metastasis *in vivo*. **(A)** Transwell invasion assay was performed in HT29 **(left)** and SW480 **(right)** cells transduced with ALKBH4 shRNA virus and control shRNA virus; *n* = 3. **(B)** Transwell invasion assay was performed in HCT116 cells transduced with ALKBH4-overexpressed virus and control-overexpressed virus; *n* = 3. **(C)** Pulmonary metastases and representative hematoxylin and eosin staining of nude mice at 13 weeks after intratumorally injected with PBS, control-overexpressed virus or ALKBH4-overexpressed virus; *n* = 8. **(D)** Summarized data on tumor lung foci in nude mice at 13 weeks in PBS, control-overexpression or ALKBH4 overexpression groups; *n* = 8. **(E)** Survival analysis was performed in nude mice bearing colorectal cancer transfected with PBS, control virus or ALKBH4 overexpression virus, respectively; *n* = 8, log-rank test.

To investigate the biological function of ALKBH4 in the metastasis of CRC, *in vitro* and *in vivo* experiments were conducted. As exhibited in the transwell invasion assays, knockdown of ALKBH4 significantly increased the invasion capability of HT29 and SW480 cells (Figure 3A). Conversely, the invasive ability was inhibited when ALKBH4 was overexpressed in HCT116 cells (Figure 3B). Furthermore, overexpression of ALKBH4 dramatically reduced lung metastasis in metastatic tumor model (Figures 3C,D). Notably, mice inoculated with ALKBH4-upregulated CRC cells had a longer overall survival time than the control groups (Figure 3E). Collectively, these results suggest that upregulation of ALKBH4 can inhibit the invasive ability of CRC cells *in vitro* and the metastatic capacity *in vivo*.

### ALKBH4 Decreases Histone H3K4me3 Modification by Interacting With WDR5

**FIGURE 4:**
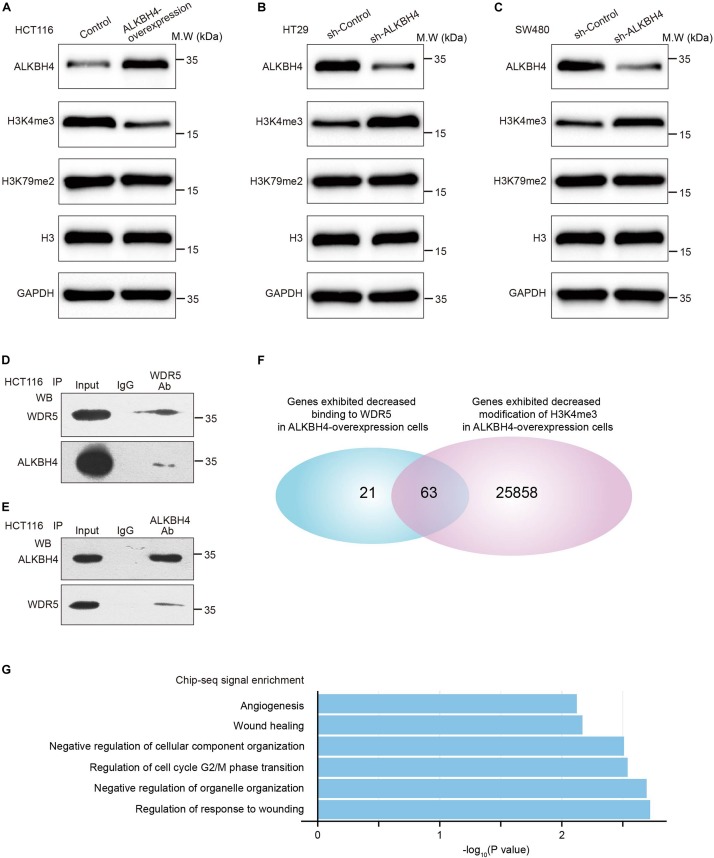
ALKBH4 decreases histone H3K4me3 modification by interacting with WDR5. **(A)** Immunoblot of ALKBH4, H3K4me3, and H3K79me2 in HCT116 cells transduced with ALKBH4-overexpressed virus and control virus. H3 and GAPDH were used as loading controls. **(B,C)** Immunoblot of ALKBH4, H3K4me3, and H3K79me2 in HT29 **(B)** and SW480 **(C)** cells transduced with sh-ALKBH4 and sh-Control virus. H3 and GAPDH were used as loading controls. **(D,E)** coimmunoprecipitation detected the interaction of WDR5 and ALKBH4 in the HCT116 cells. The input and WDR5 **(D)** or ALKBH4 **(E)** immunoprecipitates were separated by SDS-PAGE. The specific immunoprecipitation of WDR5 and ALKBH4 was confirmed by Western blot. **(F)** Venn diagram shows 63 genes with both decreased binding to WDR5 and reduced H3K4me3 modification in ALKBH4 overexpressed cells. **(G)** GO enrichment analysis of 63 overlapping genes.

We further explore the mechanism by which ALKBH4 repressed EMT and metastasis in CRC. ALKBH4 belongs to the AlkB family of non-heme Fe (II)/a-ketoglutarate-dependent dioxygenases, whose function has been implicated in the repair of methylation damage in DNA and RNA (Lee et al., 2005). To determine whether the downregulated level of ALKBH4 in CRC cells may promote the alteration in methylation level, Western blot assay was performed to detect the epigenetic modification. The assay demonstrated that overexpression of ALKBH4 resulted in the significant reduction of H3K4me3, but not H3K79me2 (Figure 4A). Downregulation of ALKBH4 significantly increased the expression of H3K4me3, but not H3K79me2 (Figures 4B,C). Given that WDR5 is a methyltransferase of H3K4me3 (Dou et al., 2006; van Nuland et al., 2013), we speculated the possibility of the interaction between ALKBH4 and WDR5. Co-IP (Co-Immunoprecipitation) assay was then conducted and the results showed that WDR5 and ALKBH4 interacted with each other in HCT116 cells (Figures 4D,E). The results indicated that ALKBH4 may regulate histone H3K4me3 modification through interacting with WDR5. To address whether ALKBH4 modulates WDR5 and H3K4me3 genomic binding genome-wide, we performed chromatin immunoprecipitation coupled with high-throughput sequencing (ChIP-seq) for WDR5 and H3K4me3 in ALKBH4-upregulated and control HCT116 cells. ChIP-seq analysis showed that 84 genes exhibited decreased binding to WDR5 after overexpression of ALKBH4 (Figure 4F). Furthermore, upregulation of ALKBH4 led to a decrease in H3K4me3 modification of 25,921 gene promoters (Figure 4F). Sixty-three genes showed both decreased binding to WDR5 and reduced H3K4me3 modification in ALKBH4-overexpressed cells (Figure 4F). Histone H3K4me3 modification is a hallmark for transcription initiation and associated with active gene transcription (Zhang et al., 2016; Batie et al., 2019). The GO analysis showed that WDR5 binding efficiency and H3K4me3 modification level were significantly decreased in the promoter region of those genes, which may regulate EMT and wound healing, after transduction of ALKBH4 overexpression virus in HCT116 cells (Figure 4G). The data suggested that ALKBH4 decreased histone H3K4me3 modification by competitively binding to WDR5 and suppressed EMT and metastasis in CRC.

### Downregulation of ALKBH4 Promotes EMT Progression in CRC via miR-21

**FIGURE 5:**
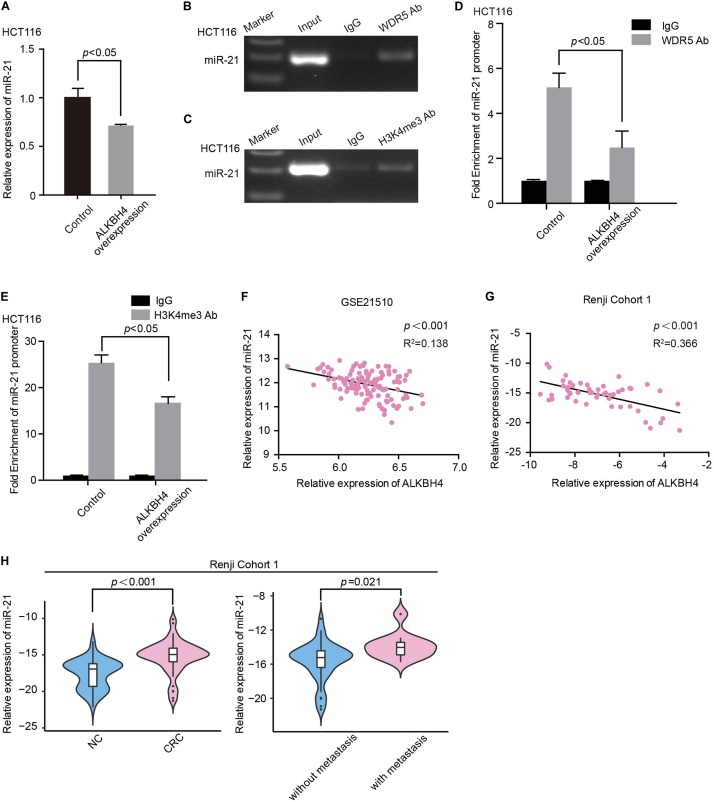
ALKBH4 inhibits miR-21 by decreasing H3K4me3 modification in the promoter region. **(A)** Real-time PCR result of miR-21 expression after transduction of ALKBH4 overexpression adenovirus in HCT116 cells; *n* = 3, non-parametric Mann–Whitney test. **(B,C)** miR-21 was detected in the chromatin sample immunoprecipitated from HCT116 cells using an antibody against WDR5 **(B)** or H3K4me3 **(C)**. **(D,E)** Real-time PCR of the ChIP samples shows the binding efficiency of WDR5 **(D)** or H3K4me3 **(E)** to the miR-21 promoter after transduction of ALKBH4 overexpression adenovirus in HCT116 cells; *n* = 3, non-parametric Mann–Whitney test. **(F,G)** The correlation between the relative expression of ALKBH4 and miR-21 in dataset GSE21510 **(F)** and Renji Cohort 1 **(G)**. **(H)** The relative expression of miR-21 was detected in CRC tissues from patients with or without metastasis and adjacent non-tumor colorectal tissues (Renji Cohort 1), non-parametric Mann–Whitney test. Error bars in the scatter plots represent SEM.

Among the overlapping genes, we discovered miR-21, which has been considered as a representative oncogenic miRNA and also associated with EMT and a poor prognosis in CRC (Yang et al., 2017), might be an essential downstream target. Overexpression of ALKBH4 significantly decreased the expression of miR-21 in HCT116 cells (Figure 5A). The ChIP-PCR data further indicated that WDR5 and H3K4me3 directly bound to the promoter region of miR-21 (Figures 5B,C). Moreover, overexpression of ALKBH4 resulted in a decrease in the binding efficiency of WDR5 and the modification level of H3K4me3 in miR-21 promoter (Figures 5D,E). In addition, real-time PCR data showed there was a significantly negative correlation between ALKBH4 and miR-21 both in microarray dataset (GSE21510) from GEO database and Renji Cohort1 (Figures 5F,G). MiR-21 was significantly increased in CRC tissues compared with paired adjacent non-tumor tissues from patients in Renji Cohort 1, especially in patients with metastasis (Figure 5H). Furthermore, real-time PCR data showed that down-regulation of WDR5 increased the expression of E-cadherin and ZO-1, and decreased the expression of miR-21, N-cadherin and Fibronection in HCT116 cells (Supplementary Figures S2A,B). These data indicated that overexpression of ALKBH4 inhibits the expression of miR-21 by decreasing H3K4me3 modification in the promoter region.

**FIGURE 6:**
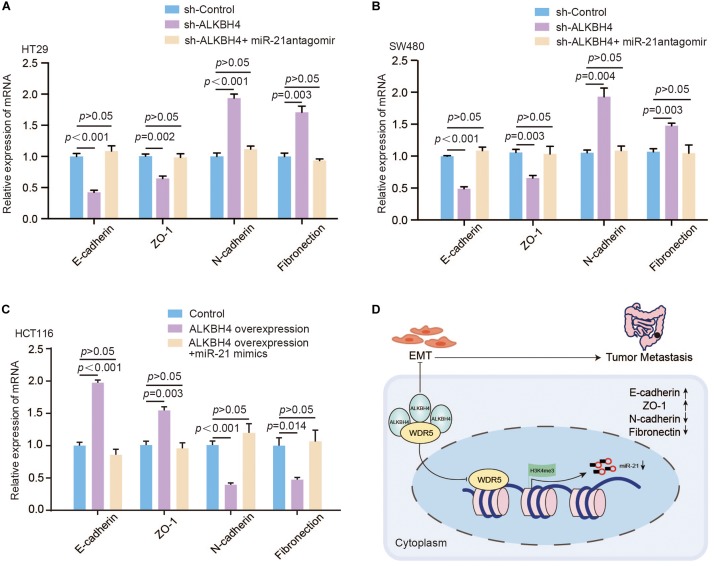
ALKBH4 regulates EMT through modulating the expression of miR-21. **(A,B)** Real-time PCR results of molecular markers of EMT in HT29 **(A)** or SW480 **(B)** cells after sh-ALKBH4 virus and/or miR-21 antagomir treatment; *n* = 3, non-parametric Mann–Whitney test. **(C)** Real-time PCR results of molecular markers of EMT in HCT116 cells after overexpression of ALKBH4 and miR-21; *n* = 3, non-parametric Mann–Whitney test. **(D)** A schematic model of ALKBH4 functions in the metastasis of CRC. ALKBH4 may competitively bound WDR5 and decreased histone H3K4me3 modification on miR-21 promoter and eventually prohibited EMT progression in the metastasis of CRC. Error bars in the scatter plots represent SEM.

Furthermore, to validate whether ALKBH4 inhibits EMT progress via decreasing miR-21, we performed rescue experiments. Real-time PCR data demonstrated that the decreased expression of E-cadherin and ZO-1 and increased expression of N-cadherin and Fibronection caused by downregulation of ALKBH4 could be partially reversed by knocking down of miR-21 (Figures 6A,B). Meanwhile, upregulation of miR-21 significantly reversed the increased expression of E-cadherin and ZO-1 and decreased expression of N-cadherin and Fibronection in ALKBH4-upregulated cells (Figure 6C).

Taken together, ALKBH4 competitively bound WDR5 and decreased histone H3K4me3 modification on miR-21 promoter and eventually prohibited EMT progression in CRC (Figure 6D).

## Discussion

Metastasis of tumor remains one of the most urgent and poorly addressed challenges in cancer therapy (Fidler and Kripke, 2015). Epithelial-Mesenchymal Transition and its associated cellular changes have been implicated to play a critical role in the metastasis process (Dongre and Weinberg, 2019), yet the interplay between the epigenetic regulatory mechanisms and EMT remains largely unknown. Using a high-content screen with a shRNA library covering 384 genes involved in modulating epigenetic modification, we have identified that ALKBH4 regulates cellular EMT process in a suppressive way. In cultured CRC cells and mouse tumor metastasis models, overexpression of ALKBH4 markedly inhibits cell migration *in vitro* and metastasis *in vivo*. Accordingly, our results consistently point to the notion that ALKBH4 serves as an essential regulator in inhibiting metastasis of CRC.

During the carcinogenesis in CRC, major cellular functions and pathways, including potential to repair DNA damage, are dysregulated (AlDubayan et al., 2018). ALKBH4 belongs to the AlkB family of non-heme Fe (II)/a-ketoglutarate-dependent dioxygenases, whose function have been implicated in the repair of methylation damage in DNA and RNA (Lee et al., 2005). Available evidences also indicate that several members of AlkB family, such as ALKBH3 and ALKBH5, are closely linked to the inhibition of tumorigenesis and progression in various human cancers (Stefansson et al., 2017; Wang et al., 2018; Zhu et al., 2019). ALKBH4 has been previously illustrated in mediating demethylation of a monomethylated site in actin and depletion of ALKBH4 contributes to defects in cytokinesis and cell motility (Li et al., 2013), however, the underlying molecular mechanisms of ALKBH4 in tumor remain unknown. In our study, ALKBH4 markedly suppressed EMT and CO-IP assay demonstrated that ALKBH4 directly interacted with WDR5 in CRC cells, a key component of histone methyltransferase complex, along with the decreased level of H3K4me3 histone modification (Dou et al., 2006; van Nuland et al., 2013). The previous studies have shown that WDR5 is a major driver of cell progression in various cancer types (Kim et al., 2014; Chen et al., 2015; Dai et al., 2015) and its functional mechanism has been elucidated in CRC by triggering EMT process in response to the PI3K/AKT signaling pathway (Tan et al., 2017). As a consequence, we inferred that ALKBH4 may competitively bind to WDR5, and thus decrease H3K4me3 histone modification on the target genes. It has been documented that aberrant epigenetic signatures are associated with cancer metastasis and deficiency of H3K4 methyltransferase extends the life span in Caenorhabditis elegans (Greer et al., 2010),which are in accordance with our results from a mechanistic perspective.

When we explored the mechanism by which ALKBH4 contributes to the inhibition in metastasis of CRC. We discovered the involvement of miR-21, which is a major player involved in tumor initiation, progression and metastasis of various types of cancers (Frankel et al., 2008; Zhu et al., 2008; Qi et al., 2009; Tian et al., 2019), including CRC (Yang et al., 2017), and considered as a representative oncogenic miRNA. Interestingly, EMT can be inhibited by down-regulating miR-21 expression in breast cancer cell (Du et al., 2019). These observations are consistent with our findings that ALKBH4 suppresses the expression of miR-21 by decreasing H3K4me3 modification in the promoter region and eventually inhibits EMT in CRC cells.

In summary, our study elucidates the mechanism of ALKBH4 in inhibiting the EMT and metastasis of CRC. These findings add diverse roles and mechanistic insight into our understanding of the interplay between epigenetic factors and EMT progression, defining ALKBH4 as a potential prognostic biomarker for the prevention of cancer cell invasion and metastatic spread.

## Data Availability Statement

The authors declare that all data supporting the conclusions of this study are presented within the article and the Supplementary Material and are available from the authors.

## Ethics Statement

The studies involving human participants were reviewed and approved by the Ethics Committee of Renji Hospital, Shanghai Jiao Tong University School of Medicine. The patients/participants provided their written informed consent to participate in this study. The animal study was reviewed and approved by the Ethics Committee of Renji Hospital, Shanghai Jiao Tong University School of Medicine.

## Author Contributions

CS, TY, TT, DS, LR, YZ, XZha, YC, YY, YM, XZhu, and XT performed the experiments and analyzed data. J-YF and LJ provided the clinical specimen. HC conducted the data analysis. JH and BX conceived and wrote the manuscript and supervised the study.

## Conflict of Interest

The authors declare that the research was conducted in the absence of any commercial or financial relationships that could be construed as a potential conflict of interest.

## Footnotes

1 http://www.ncbi.nlm.nih.gov/geo/

2 https://pypi.org/project/MACS2/

3 http://tophat.cbcb.umd.edu

4 http://cran.r-project.org/bin/windows/base/old/3.0.2/
