# Supplementary material for: ALKBH4 Functions as a Suppressor of Colorectal Cancer Metastasis via Competitively Binding to WDR5
Source: Front Cell Dev Biol. 2020 May 14;8:293. doi: 10.3389/fcell.2020.00293 (PMC7240015; doi:10.3389/fcell.2020.00293)
Supplement: Supplementary file 1 [file Data_Sheet_1.DOCX]

**Supplementary Figure S1. The relative expression of ALKBH4 in CRC cell lines. A,** Real-time PCR data showed the relative expression of ALKBH4 in CRC cell lines; n=3.

**Supplementary Figure S2. Downregulation of WDR5 suppressed the expression of miR-21 and inhibited EMT progress. A,** The knockdown efficiency of WDR5 siRNA was evaluated in HCT116 cells; n=3, nonparametric Mann–Whitney test. **B,** The expression of mir-21, E-cadherin, ZO-1, N-cadherin and Fibronection were detected in HCT116 cells after transfection of WDR5 siRNA; n=3, nonparametric Mann–Whitney test. Error bars in the scatter plots represent SEM.
