# Supplementary figures and images for: ALKBH4 Functions as a Suppressor of Colorectal Cancer Metastasis via Competitively Binding to WDR5
Source: Front Cell Dev Biol. 2020 May 14;8:293. doi: 10.3389/fcell.2020.00293 (PMC7240015; doi:10.3389/fcell.2020.00293)

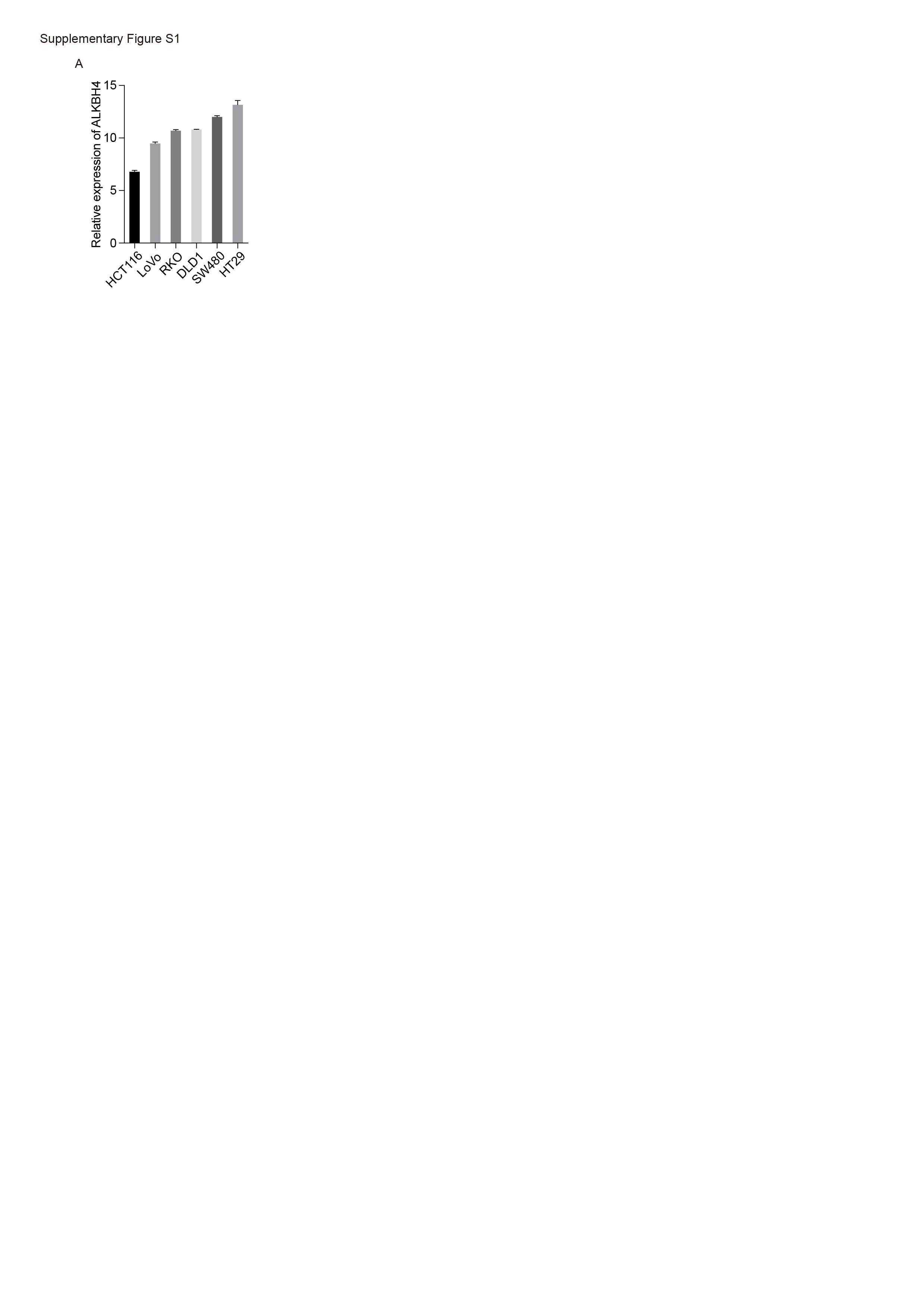

Supplement: Supplementary file 5 [file Image_1.JPEG]

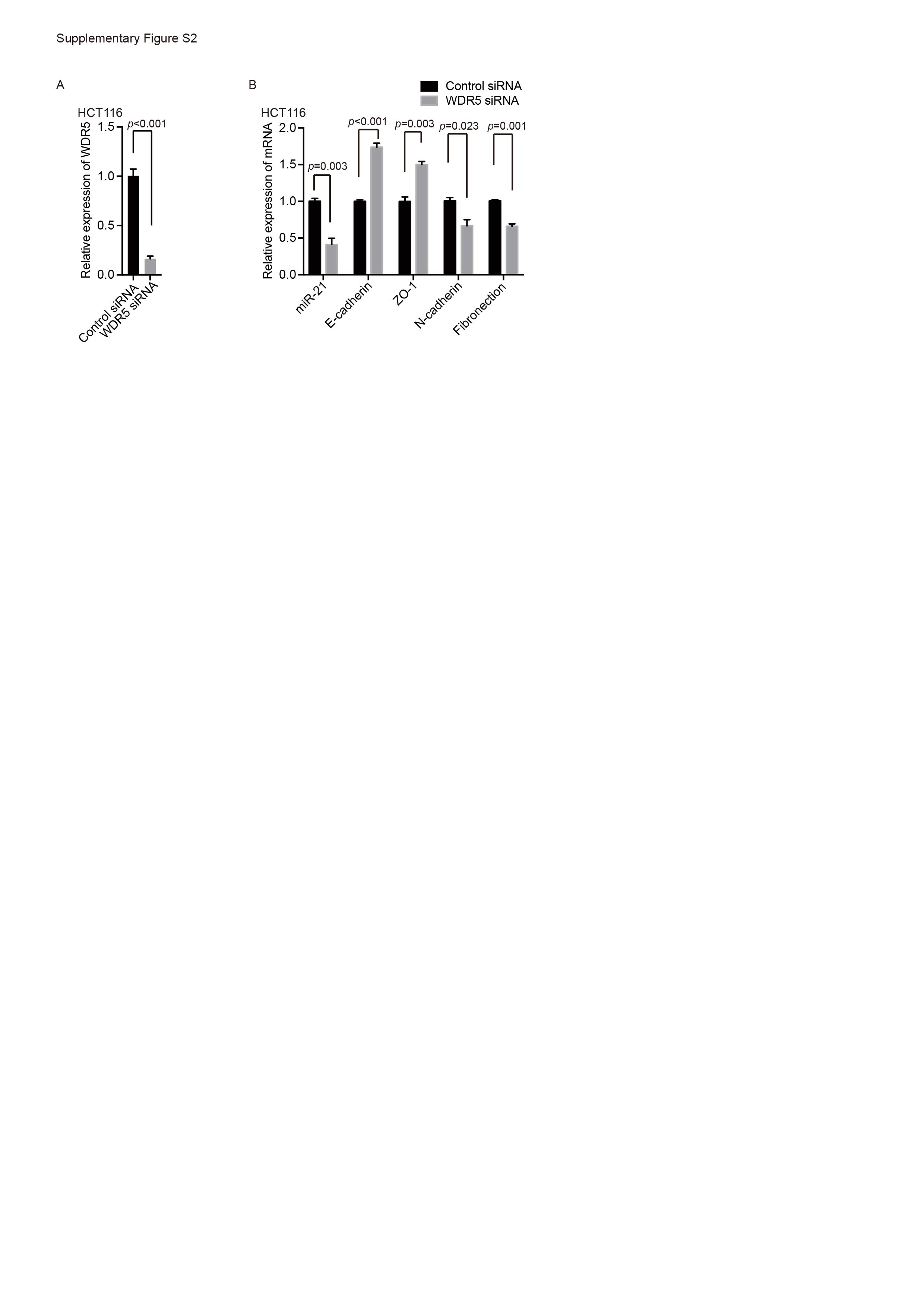

Supplement: Supplementary file 6 [file Image_2.JPEG]
